# Optimization and Pharmacological Validation of a Leukocyte Migration Assay in Zebrafish Larvae for the Rapid *In Vivo* Bioactivity Analysis of Anti-Inflammatory Secondary Metabolites

**DOI:** 10.1371/journal.pone.0075404

**Published:** 2013-10-04

**Authors:** María Lorena Cordero-Maldonado, Dany Siverio-Mota, Liliana Vicet-Muro, Isabel María Wilches-Arizábala, Camila V. Esguerra, Peter A. M. de Witte, Alexander D. Crawford

**Affiliations:** 1 Laboratory for Molecular Biodiscovery, Department of Pharmaceutical and Pharmacological Sciences, University of Leuven, Leuven, Belgium; 2 Faculty of Chemical Sciences, School of Biochemistry and Pharmacy, University of Cuenca, Cuenca, Ecuador; 3 Department of Pharmacy, Faculty of Chemistry-Pharmacy, Central University “Marta Abreu” of Las Villas, Santa Clara, Cuba; 4 Luxembourg Centre for Systems Biomedicine, University of Luxembourg, Esch-sur-Alzette, Luxembourg; National University of Singapore, Singapore

## Abstract

Over the past decade, zebrafish (*Danio rerio*) have emerged as an attractive model for *in vivo* drug discovery. In this study, we explore the suitability of zebrafish larvae to rapidly evaluate the anti-inflammatory activity of natural products (NPs) and medicinal plants used in traditional medicine for the treatment of inflammatory disorders. First, we optimized a zebrafish assay for leukocyte migration. Inflammation was induced in four days post-fertilization (dpf) zebrafish larvae by tail transection and co-incubation with bacterial lipopolysaccharides (LPS), resulting in a robust recruitment of leukocytes to the zone of injury. Migrating zebrafish leukocytes were detected *in situ* by myeloperoxidase (MPO) staining, and anti-inflammatory activity was semi-quantitatively scored using a standardized scale of relative leukocyte migration (RLM). Pharmacological validation of this optimized assay was performed with a panel of anti-inflammatory drugs, demonstrating a concentration-responsive inhibition of leukocyte migration for both steroidal and non-steroidal anti-inflammatory drugs (SAIDs and NSAIDs). Subsequently, we evaluated the bioactivity of structurally diverse NPs with well-documented anti-inflammatory properties. Finally, we further used this zebrafish-based assay to quantify the anti-inflammatory activity in the aqueous and methanolic extracts of several medicinal plants. Our results indicate the suitability of this LPS-enhanced leukocyte migration assay in zebrafish larvae as a front-line screening platform in NP discovery, including for the bioassay-guided isolation of anti-inflammatory secondary metabolites from complex NP extracts.

## Introduction

Beginning with Hippocrates’ prescription of willow bark extract for the relief of *calor*, *dolor, tumor* and *rubor* – the four cardinal signs of inflammation – the search for novel, more efficient, and safer anti-inflammatories remains an ongoing challenge in drug discovery. Over time, even with the clear success of aspirin and other non-steroidal anti-inflammatory drugs (NSAIDs), the classic gastrointestinal and cardiovascular side effects remain a major drawback from these therapies [1]–[3]. Because of the large number of bioactive, structurally diverse secondary metabolites found in nature, small molecules from natural sources represent an attractive alternative for anti-inflammatory drug discovery [4]. This benefit, in combination with the rise in popularity of zebrafish (*Danio rerio* – Cyprinidae) as an *in vivo* model to study inflammation becomes an excellent strategy for the identification of novel compounds.

Zebrafish have become a good model organism to study hematopoiesis, which during embryonic development is characterized by primitive erythro- and myelopoiesis occurring in the intermediate cell mass and in the anterior lateral mesoderm (ALM), respectively [5]. During somite stages (as early as 11 hours post-fertilization, or hpf), zebrafish embryos express a myeloid-specific transcription factor (*pu.1*), which directs the production and maturation of granulocytes and macrophages in the ALM. Later in development, from 26 hpf onwards, primitive macrophages actively migrate to engulf apoptotic cells. Subsequently a transient wave of myelopoiesis shifts to the caudal region of the embryo, which is followed by definitive myelopoiesis in the kidney at 3 days post-fertilization (dpf) [5]. Essential information about myeloid cells and their efficient participation in defending an injured host has also been revealed by studying inflammatory-induced migration and resolution in zebrafish [6]–[10].

Similar to mammals, zebrafish have three granulocyte lineages (*heterophils*, *eosinophils*, and *basophils*/*mast cells*), which constitute a potent innate immune system as demonstrated by robust migration to a site of injury or inflammation [11]–[13]. Equivalent to human neutrophils, *heterophils* are actively implicated in acute inflammation and express myeloperoxidase (MPO), a granulocyte-specific marker. Also MPO-positive, *eosinophils* and *basophils* have a functional role that is still less clear [5], [12], [14]. Moreover, migrating immature *macrophages* have been recognized at 14–16 hpf in the rostral region and at 22–24 hpf across the yolk sac. Mature *macrophages* circulate throughout the embryo and at 48 hpf they have been shown to migrate to sites infected with bacteria [5], [12], [13], [15], [16].

Additionally, key advantages of zebrafish include their high fecundity and the small size, rapid *ex utero* development, and optical transparency of embryos and larvae, with which most experiments are easily performed [5], [17]. Also, only small amounts of compounds, which are simply added to the surrounding medium and absorbed through either gastrointestinal tract or across their skin, are required for the rapid assessment of pharmacological activity *in vivo* – a unique feature among vertebrate animal models [18], [19].

Based on the principle of generating a mechanical injury to attract leukocytes to damaged zones, zebrafish embryos and larvae have proven to be suitable for the investigation of the kinetics of inflammation *in vivo* [7], [8], [12], [20]. Transgenic zebrafish strains expressing fluorescent proteins in leukocytes have allowed not only direct *in vivo* visualization of the inflammatory response [6], [8], [10] but also evaluation of the pharmacological activity of anti-inflammatory candidates in high-throughput screens for the search of new immunomodulatory lead compounds [21]. These transgenic reporter lines clearly present important advantages over non-transgenic zebrafish assays for the assessment of anti-inflammatory activity. However, their requirement for advanced imaging equipment and software may hinder their more wide-spread application, especially in laboratories with limited financial resources. For these reasons, and considering that existing zebrafish inflammation assays based on mechanical injury have not been thoroughly evaluated to date using known anti-inflammatory drugs, we considered the improvement and evaluation of a histologically-based tail injury model in zebrafish larvae as an attractive strategy to establish a cost-effective yet robust front-line screening platform for anti-inflammatory natural product discovery.

In order to establish an *in vivo* bioassay suitable for the screening and bio-guided analysis of small molecules with potential anti-inflammatory properties, we first sought to optimize an assay for acute inflammation in zebrafish larvae at 4 dpf. This method is based on the rationale that a robust, dual activation of the innate immune response triggered by mechanical and biological assaults – complete tail transection and co-incubation with lipopolysaccharides (LPS), respectively – would be sufficient to rapidly provide accurate initial information on the possible anti-inflammatory activity of compounds in question, which can be further studied as potential drug candidates.

We next pharmacologically validated this acute inflammation assay using a panel of anti-inflammatory drugs – including both steroidal and non-steroidal anti-inflammatory drugs (SAIDs and NSAIDs). Finally, we used this assay for the analysis of natural products (NPs) with well-documented anti-inflammatory activity, and to screen plant extracts (PEs) for the evaluation of their anti-inflammatory effects. The data generated with these different sets of experiments suggest that the zebrafish-based acute leukocyte migration assay could be useful as a front-line screening tool to identify novel anti-inflammatory small molecules. Moreover, this assay is particularly amenable to carry out the bioassay-guided isolation of anti-inflammatory secondary metabolites present in crude natural product extracts, as recently described in a related study [22].

## Materials and Methods

### Ethics Statement

All animal procedures were performed in accordance with Belgian and European laws, guidelines and policies for animal experimentation, housing and care (Belgian Royal Decree of 6 April 2010 and European Directive 2010/63/EU on the protection of animals used for scientific purposes of 20 October 2010). This project was approved by the institutional Ethical Committee for Animal Experimentation of the University of Leuven (approval number P101/2010).

### Animals

Adult zebrafish and embryos were raised and maintained in our facility according to standard protocols [23]. The zebrafish strains used in this study were: *fli-1*:eGFP (Ekwill background) and *nacre* (*nac^w2^*, AB background). Embryos were obtained by natural spawning and after collection and sorting, fertilized embryos were reared in 0.3 X Danieau’s medium (17.4 mM NaCl, 0.21 mM KCl, 0.12 mM MgSO_4_, 0.18 mM Ca(NO_3_)_2_, 1.5 mM HEPES pH 7.6 and 0.03 M methylene blue) at 28°C (±0.5°C). One day after fertilization *fli-1*:eGFP larvae were exposed to 0.2 mM 1-phenyl-2-thiourea (PTU; Sigma-Aldrich) to suppress melanization, while no exposure was needed for the *nacre* larvae. Medium exchange was done during the next days to ensure the viability of embryos/larvae for the assays. Four days post-fertilization (4 dpf) zebrafish larvae subjected to tail cut were first anesthetized by immersion in 0.3 X Danieau’s medium containing 70 µg/ml tricaine (ethyl 3-aminobenzoate, Sigma-Aldrich).

### Solutions

Lipopolysaccharides from *Salmonella typhosa* ATCC 10749 (10 µg/ml, LPS, Sigma-Aldrich) were reconstituted in deionized water (Milli-Q®), aliquotted in small vials and kept at −20°C until needed for the assay to stimulate leukocyte migration in larvae. Stocks (10 mM) of the SAIDs and NSAIDs dexamethasone (Sigma-Aldrich), diclofenac (Voltaren® IM/IV 75 mg/3 ml, Novartis), indomethacin (Indocid® IV 1 mg, Ovation), piroxicam (Piroxicam-ratiopharm 20 IM 20 mg, Rathipharm), prednisolone (Sigma-Aldrich) and non-anti-inflammatory drugs (non-AIDs) amantadine hydrochloride (Sigma-Aldrich), doxepin hydrochloride (Sigma-Aldrich) and melamine (Acros Organics) were prepared either in 100% dimethyl sulphoxide (DMSO) or deionized water, depending on compound solubility. Stocks (50 mM) of the NPs apigenin, (−)-α-bisabolol, caffeic acid, harpagoside, luteolin, naringenin and quercetin (Sigma-Aldrich) were prepared in 100% DMSO and testing concentrations were prepared on 0.3 X Danieau’s medium maintaining maximum 1% DMSO.

### Plant Extracts

To prepare the extracts, plants used in our study were: *Arnica montana* L. (Asteraceae), *Calendula officinalis* L. (Asteraceae), *Chamaemelum nobile* L. (Asteraceae), *Harpagophytum procumbens* DC. ex Meisn. (Pedaliaceae), *Matricaria recutita* L. (Asteraceae), *Capraria biflora* L. (Scrophulariaceae), *Syzygium montanum* (Myrtaceae), and *Schefflera chimbuensis* (Araliaceae).

#### Plant material

Dry material of *Arnica montana* (flowers), *Calendula officinalis* (petals), *Chamaemelum nobile* (flowers), *Harpagophytum procumbens* (roots) were purchased from Pharmaflore S.A. (Belgium). Aerial parts of *Capraria biflora* were collected in its natural habitat in the Botanical Garden at the Central University of Las Villas, Santa Clara (Cuba) and a voucher specimen (No. 08132) was deposited in the herbarium of the Botanical Garden. *Syzygium montanum*, and *Schefflera chimbuensis* are part of the Strathclyde Natural Products Library (SNPL) and were available for the study through collaboration with the Strathclyde Institute for Drug Research (SIDR) at the University of Glasgow and the Scottish Universities Life Science Alliance (SULSA).

#### Preparation of the extracts

Powdered dry material (each ∼30 g) of *Arnica montana* and *Calendula officinalis* were extracted with methanol (1/10 ratio) by maceration at room temperature (x 3). Powdered dry material of *Chamaemelum nobile*, *Capraria biflora,* and *Harpagophytum procumbens* (each ∼30 g) were extracted with distilled water (1/10 ratio) for one hour at 100°C (x 3). Each extract was evaporated to dryness in vacuum not exceeding 40°C, kept at 4°C, and protected from light until use. The essential oil of *Matricaria recutita* was purchased from Sjånkara (Belgium) with lot No. 1176.813. Dry methanolic extracts of *Syzygium montanum* and *Schefflera chimbuensis* were provided by SNPL.

### Toxicological Evaluation

For the toxicological analysis, ten 4 dpf zebrafish larvae were used per well in 24-well culture plates. Incubation of each set of larvae at 28°C (±0.5) was done with SAIDs, NSAIDs and non-AIDs at concentrations ranging from 0.1 µM to 500 µM, with NPs at concentrations ranging from 1 µM to 500 µM, and with PEs at concentrations ranging from 1 ug/ml to 1 mg/ml. Controls containing only vehicle (1% DMSO) and untreated larvae were processed in parallel. Examination of each set of larvae was done under light microscopy (Carl Zeiss Stemi 2000C) each hour during a period of 8 h, and a final evaluation at 24 h post-incubation. At 8 h post-incubation, signs of toxicity, e.g. pericardial oedema, cardiovascular defects (arrhythmia or decreased circulation), balance defects (loss of normal posture), locomotor defects (decreased touch response), and ultimately death were scored for every drug, NP, and PE to further establish their maximum tolerated concentration (MTC). The MTC was defined as the concentration at which not more than 2 out of 10 larvae exhibited any of the signs of toxicity after 8 h post-incubation. In this context, only non-toxic concentrations (below the MTC) were considered for further leukocyte migration analysis. All the toxicological assays were performed in duplicate using a different batch of zebrafish larvae for each set of experiments.

### Leukocyte Migration Assay

#### LPS-Enhanced leukocyte migration assay

The lipopolysaccharide- (LPS−)enhanced leukocyte migration assay was carried out by triplicate for each sample in 4 dpf zebrafish larvae. The total incubation period of the assay was eight hours. Initially, a pre-incubation step (i.e. prior to tail transection) of one hour at 28°C (±0.5°C) was carried out in 24-well culture plates. Ten larvae per well were incubated in 1 ml of testing mix, consisting of a specific concentration of each sample and the amount of 0.3 X Danieau’s medium necessary to complete 1 ml. Immediately after pre-incubation, complete tail transection in each larva was performed. Zebrafish larvae were first anesthetized by immersion in 0.3 X Danieau’s medium containing 70 µg/ml tricaine (ethyl 3-aminobenzoate, Sigma-Aldrich) and then tail transection near the tip was performed with a sterile scalpel under light microscopy (Carl Zeiss Stemi 2000C). Larvae were then placed in fresh 0.3 X Danieau’s medium without anesthetic for a few minutes until the final incubation period. For the last step, a new testing mix was prepared including 10 µg/ml lipopolysaccharides from *Salmonella typhosa* ATCC 10749 (LPS, Sigma-Aldrich Chemical) to enhance leukocyte migration to the injured zone. The final incubation step (i.e. subsequent to tail transection) of the injured larvae in contact with the pro-inflammatory agent and with the specific concentration of each SAID, NSAID, non-AID, NP, and PE at 28°C (±0.5°C) was done for 7 h. Within this period, sporadic evaluations of the larvae were done to examine their viability during the time of the assay. Larvae that completed the incubation time were fixed overnight in 4% formaldehyde (Sigma-Aldrich) at 4°C. Controls containing only the vehicle (1% DMSO) were processed in parallel.

#### Cytochemical myeloperoxidase staining

Fixed larvae were gently washed twice with 1 X PBST (PBS-1X phosphate buffered saline, Gibco +0.1% Tween 20) for 5–10 min and then incubated at room temperature in 1 ml of freshly prepared Leucognost® Pox (Merck) staining solution, made according the directions of the provider. Observation of stained black-brown cells (leukocytes) in the larvae was possible within 15 min of incubation. Evaluation of the migrating leukocytes, particularly surrounding the injured region, in each larva was done under light microscopy (Carl Zeiss Stemi 2000C) and scoring was done using a migration scale established to semi-quantify the amount of migrating leukocytes on one side of each larva.

#### Relative leukocyte migration calculation

The average of the value achieved for each of the ten larvae tested in triplicate for one sample is expressed as relative leukocyte migration (RLM) according to the formula: RLM = leukocyte migration of the larva/average leukocyte migration of the negative control. The average of the calculated RLM for each sample is plotted against the control (1% DMSO) and for the interpretation of the results, RLM ≤0.5 is considered as cutoff point for a significant anti-inflammatory activity.

### Imaging

Zebrafish larvae were screened for leukocyte migration using a Stemi 2000C stereo microscope (2.0 X) from Carl Zeiss. Micrographs of the stained tails of zebrafish larvae were taken on a MZ10F stereo microscope (1.0 X) from Leica equipped with a DFC310 FX digital camera using Leica Application Suite version 3.6.0 (Build: 488) and processed with Microsoft Office Picture Manager and Adobe Photoshop software.

### Statistical Analysis

All leukocyte migration experiments were performed in side-by-side triplicates, using ten larvae for each condition. Statistical analysis (GraphPad Prism 5 software) was performed using one-way analysis of variance (ANOVA) followed by Dunnetts’s test for multiple comparisons. Unpaired *t*-test with Welch’s correction was used for single comparisons of each group with the control 1% DMSO+LPS to determine statistical significance. The criterion for statistical significance was p<0.05 (***p<0.001, **0.001<p<0.01, *0.01<p<0.05). Error bars on all graphs represent the standard error of the mean (SEM).

## Results

### *in vivo* Toxicological Evaluation

Toxicological assessment of all the samples analyzed in our study was done prior to the evaluation of their anti-inflammatory properties. The maximum tolerated concentrations (MTCs) were determined at 8 h post-treatment according to toxicological parameters including pericardial oedema, cardiovascular defects (arrhythmia or decreased circulation), balance defects (loss of normal posture), locomotor defects (decreased touch response), and death. Toxicity was elicited for most of the tested drugs at a concentration higher than 200 µM with the exception of piroxicam (MTC 100 µM), diclofenac (MTC 10 µM), and doxepin (MTC 100 µM). In the case of the NPs, 500 µM was the MTC for apigenin, luteolin, quercetin and caffeic acid; 300 µM for harpagoside; and 30 µM for naringenin and (−)-α-bisabolol. Maximum tolerated concentrations for PEs were variable. Low toxicity was exhibited by the aqueous extracts of *Capraria. biflora* (MTC 1 mg/ml), *Chamaemelum nobile* (MTC 700 µg/ml) and *Harpagophytum procumbens* (MTC 500 µg/ml), whereas the methanolic extract of *Schefflera chimbuensis* induced toxicity at concentrations higher than 100 µg/ml. Finally, the methanolic extracts of *Arnica montana*, *Calendula officinalis* and *Syzigium montanum* exhibited a common MTC of 3 µg/ml, and the essential oil of *Matricaria recutita* a MTC of 10 µg/ml. Vehicle-treated (1% DMSO) and untreated larvae were processed in parallel to minimize experimental variability in this toxicological evaluation.

### LPS-Enhanced Leukocyte Migration towards Tail Injury

To induce the recruitment of leukocytes to a site of injury, we performed a transection of the tail in zebrafish larvae following previously described guidelines [12]. A series of initial experiments was conducted using normally pigmented (Ekwill strain) zebrafish larvae – adequately treated with 1-phenyl-2-thiourea (PTU) to inhibit melanization [24]. This was intended to establish experimental conditions such as 1) appropriate developmental stage of zebrafish for the assay, 2) precise location of the tail transection, and 3) hours post-injury required to achieve a robust leukocyte migration response prior to detection of MPO-positive cells. Next, we sought to avoid interference with the cytochemical staining for MPO by any remaining melanocytes not inhibited by the addition of PTU, and therefore used the *nacre* strain [25], which has strongly reduced numbers of melanocytes in most tissues.

**Figure 1:**
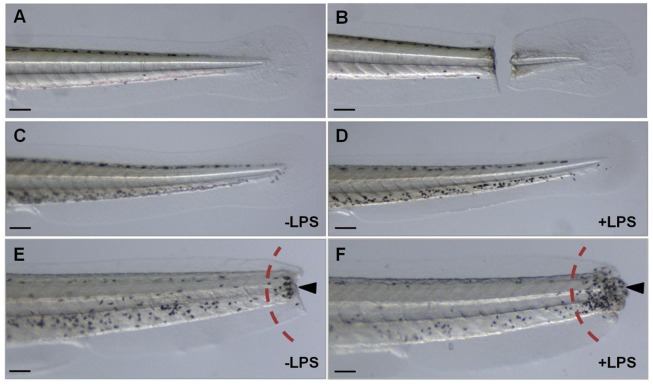
LPS-enhanced leukocyte migration assay in 4 dpf zebrafish larvae. All larvae (*nacre*) are four days post-fertilization (4 dpf), with anterior to the left, scale bar = 10 µm. **A**, tail of alive larva without tail cut; **B**, tail of alive larva with tail cut; **C–D**, whole-mount MPO staining in uncut tails of zebrafish larvae; **C**, without lipopolysaccharides (−LPS) and **D**, with lipopolysaccharides (+LPS); **E–F**, whole-mount MPO staining in cut tails of zebrafish larvae; **E,** without the inclusion of LPS; **F**, with the inclusion of LPS. Dark-spots (marked by arrows) represent the migrating leukocytes, which are semi-quantified in the region to the right of the dashed red arc.

Based on these preliminary experiments, we determined that we could obtain the most consistent and robust leukocyte migratory response if zebrafish larvae at 4 dpf were subjected to a complete tail transection made in a target region comprising 0.5 mm (±0.2 mm, tolerated range) from the tip of the tail of each larva (Figure 1A and 1B). Additionally, we took advantage of the strong immune-stimulating properties elicited by bacterial LPS in order to promote an even greater migratory response in the larvae (Figure 1C and 1D). By co-incubation with LPS we observed reproducible and robust congregations of leukocytes, which clustered mainly in the affected region in the tails (Figure 1E and 1F).

### Semi-Quantification of Migrating Leukocytes

**Figure 2:**
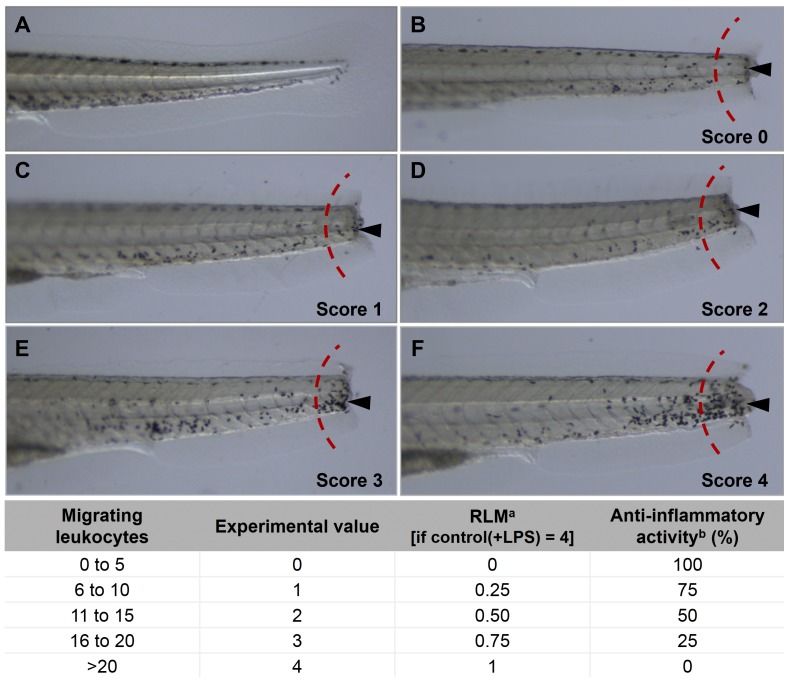
Scoring of leukocyte migration in the transected tails of zebrafish larvae. All larvae (*nacre*) are four days post-fertilization (4 dpf), with anterior to the left, scale bar = 10 µm. Dark spots (marked by arrows) represent leukocytes migrating to the injured zone in the transected tails. Migrating leukocytes were counted in the region to the right of the dashed red arc. **A**, tail of an uncut larva; **B**, tail-cut with score 0; **C**, tail-cut with score 1; **D**, tail-cut with score 2; **E** tail-cut with score 3; **F,** tail-cut with score 4. **^a^**Experimental values obtained for each larvae are normalized to a relative value expressed as relative leukocyte migration (RLM); +LPS: with inclusion of lipopolysaccharides. **^b^**Percentage of anti-inflammatory activity is obtained as (1−RLM)x100.

To determine the extent of leukocyte migration generated after exposing 4 dpf zebrafish larvae to mechanical and biological stimuli – namely tail transection and LPS exposure – we used a commercially available whole-mount cytochemical staining method (Leucognost® Pox, Merck) to detect MPO activity *in situ*. To minimize the number of handling steps post-staining, this method was chosen over the alternative myeloperoxidase stain Sudan Black B [26], [27], which also stains phospholipids, neural fats and sterols. In our experiments, we visualized migrating leukocytes in 4 dpf larvae at 8 h post-injury. Microscopic evaluations of MPO-stained larvae allowed us to score the degree of leukocyte migration at the trauma site according to a comparative scale that we established particularly for this purpose (Figure 2). Myeloperoxidase-positive cells, appearing as black-brown dots, were counted in a segment at the edge of the tail on one side of every larva (Figure 2). The number of leukocytes was then graded according to the comparative scale and subsequently converted into a relative value expressed as the relative leukocyte migration (RLM) activity for the total number or larvae for one sample. In this assay, we considered relative leukocyte migration values ≤0.5 to represent significant anti-inflammatory activity.

### Effect of Anti-Inflammatory Drugs and Non-Anti-Inflammatory Compounds in the Migration of Zebrafish Leukocytes

**Figure 3:**
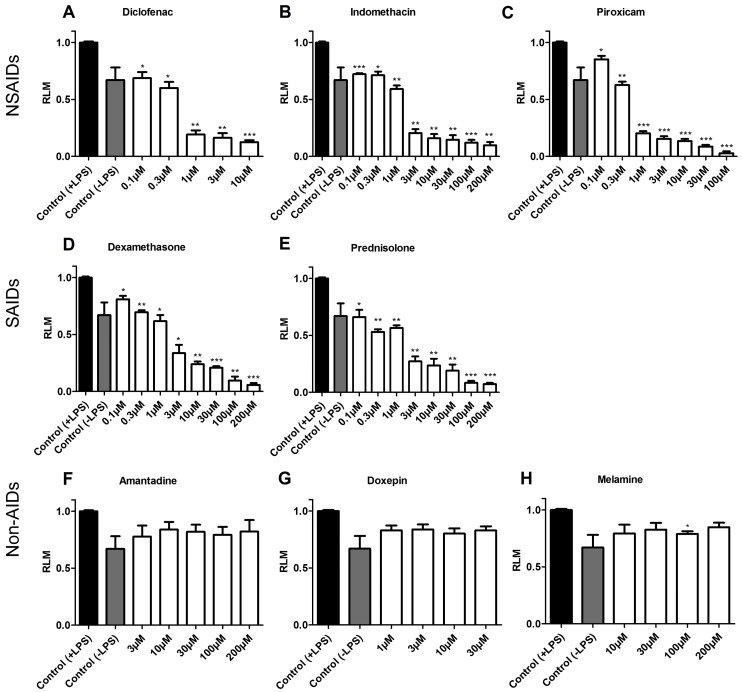
Inhibition of the leukocyte migration by known anti-inflammatory drugs and non anti-inflammatory compounds. After exposing the larvae to mechanical and biological damages, anti-inflammatory drugs and non anti-inflammatory compounds were evaluated for their activity in the LPS-enhanced leukocyte migration assay. Migrating MPO-positive cells were counted in the tail tip and the results expressed as relative leukocyte migration (RLM) values. Results for NSAIDs (upper panel) and SAIDs (middle panel) show a significant inhibition of leukocytes migration in a concentration-dependent manner while non anti-inflammatory drugs (lower panel) indicate no significant effect on leukocyte migration. In each case, values are plotted as RLM (SEM, n = 3 replicates with 10 larvae each) and a value of 0.5 was established as a cut-off for anti-inflammatory activity. ***p<0.001, **0.001<p<0.01, *0.01<p<0.05.

We conducted validation experiments using a panel of SAIDs and NSAIDs and randomly selected control compounds with no anti-inflammatory activity. As a proof of principle, the anti-inflammatory effect displayed by commercially available NSAIDs (diclofenac, indomethacin and piroxicam) and SAIDs (dexamethasone and prednisolone) exhibited statistically significant values at diverse concentrations, ranging from 0.1 µM to 200 µM (Figure 3A–E). In the LPS-enhanced leukocyte migration assay, diclofenac, indomethacin and piroxicam showed RLM values of 0.13, 0.09 and 0.03, respectively, when tested at its MTC 10, 200 and 100 µM, respectively. In contrast, the same NSAIDs evaluated at a lower concentration (0.1 µM) displayed RLM of 0.69 for diclofenac, 0.72 for indomethacin and 0.85 for piroxicam, corresponding to a low inhibition of migrating leukocytes. For the SAIDs dexamethasone and prednisolone, RLMs of 0.06 and 0.07 were achieved respectively at 200 µM whereas RLM values of 0.81 and 0.66 at 0.1 µM indicated a poor effect on the inhibition of leukocyte migration (Figure 3D–E).

As expected, the non-anti-inflammatory compounds tested in our assay – amantadine, doxepin and melamine – did not have a significant effect on the inhibition of leukocyte migration since all the tested concentrations in our experiments gave RLM values much higher than the cutoff (RLM ≤0.5). Thus, amantadine and melamine (both evaluated at their MTC 200 µM) and doxepin (MTC 30 µM) showed RLM values of 0.82, 0.85 and 0.71, respectively (Figure 3F–H).

Consequently, these results showed that the zebrafish-based leukocyte migration method was able to detect an expected anti-inflammatory activity revealed by the concentration-dependent decrease of leukocyte migration towards the injured tail region in the larvae.

### Evaluation of the Anti-Inflammatory Activity of Natural Products in the LPS-Enhanced Leukocyte Migration Assay

After pharmacologically validating the LPS-enhanced leukocyte migration assay by demonstrating a dose-response inhibition of leukocyte migration in zebrafish larvae exposed to SAIDs and NSAIDs, we next used the assay to evaluate the anti-inflammatory effect of NPs. For this purpose we used small molecules derived from plant species and with well-documented anti-inflammatory activity in other models (*in vivo* and *in vitro*), including four flavonoids (apigenin, naringenin, luteolin, and quercetin), one sesquiterpenoid ((−)-α-bisabolol)), one iridoid (harpagoside), and one phenolic compound (caffeic acid).

All flavonoids – apigenin, naringenin, luteolin, and quercetin – showed moderate but significant anti-inflammatory activity up to their MTCs. Particularly apigenin and quercetin tested at their common MTC (500 µM) resulted in RLM values of 0.35 and 0.31, respectively, corresponding to 65 and 69%, respectively, anti-inflammatory activity. Similarly, naringenin (MTC 30 µM) showed significant effects, with a RLM value of 0.37. In all cases, only weak inhibition of leukocyte migration was reached with low concentrations of the flavonoids (Table 1).

**Table 1:** Anti-inflammatory activity of natural products.

Natural product	Leukocyte migration^a^ (RLM)						Anti-inflammatory activity at MTC
	1 µM	10 µM	30 µM	100 µM	300 µM	500 µM	
Apigenin	0.74	0.53*	0.54	0.37*	0.37**	0.35***	65%
Luteolin	0.63*	0.61***	0.55*	0.48*	0.44**	0.41**	59%
Naringenin	0.67*	0.42**	0.37**	T	T	T	63%
Quercetin	0.65	0.55*	0.35*	0.42*	0.40	0.31*	69%
(−)-α-bisabolol	0.53*	0.50*	0.48	T	T	T	52%
Caffeic acid	0.80	0.71	0.59*	0.59*	0.34*	0.14**	86%
Harpagoside	0.48*	0.29*	0.30*	0.15*	0.12*	T	85%

a Leukocyte migration is expressed as relative values (RLM).

*** p<0.001,

** 0.001<p<0.01,

* 0.01<p<0.05. T: toxic concentration.

Moderate inhibition of leukocytes migration was also displayed by the sesquiterpene (−)-α-bisabolol. Relative leukocyte migration values achieved with high concentrations (0.50 at 10 µM and 0.48 at 30 µM) yet fulfilled our cut-off criteria for significant anti-inflammatory activity (≤0.5). More interestingly, in our LPS-enhanced assay the iridoid harpagoside and the phenolic compound caffeic acid inhibited the migration of leukocytes to the injured zone in a dose-response manner (Table 1). High concentrations of both NPs achieved RLM values of 0.12 (harpagoside) and 0.14 (caffeic acid), corresponding to significant anti-inflammatory activity.

### Screen for Anti-Inflammatory Activity in Plant Extracts

The LPS-enhanced leukocyte migration assay was next used as an *in vivo* tool to screen for anti-inflammatory activity in crude extracts of diverse NPs. Based on ethnomedicinal uses, six plant species from different genera were selected for evaluation of their anti-inflammatory activity in our established acute inflammation assay in zebrafish. The selection included *Arnica montana*, *Calendula officinalis*, *Chamaemelum nobile*, *Harpagophytum procumbens*, *Matricaria recutita*, and *Capraria biflora*. Moreover, two randomly chosen species – *Syzygium montanum* and *Schefflera chimbuensis*, both without previous ethnopharmacological data – were also included in the study. However, after initial *in vivo* toxicological evaluation, *A. montana*, *C. officinalis*, *M. recutita*, and *S. montanum* were left out of the screen due to their toxicity effects.

**Figure 4:**
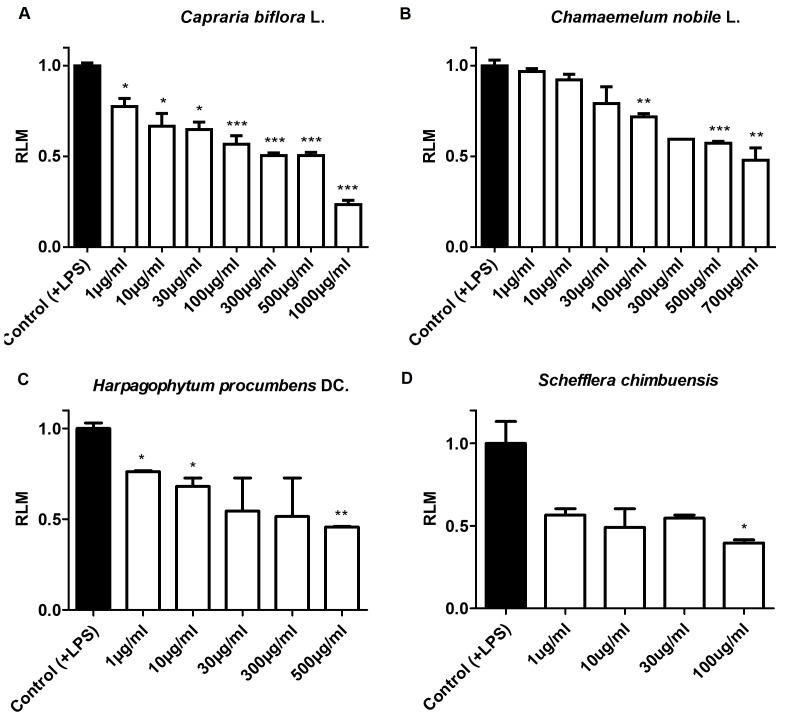
Anti-inflammatory effect of aqueous and methanolic plan extracts. Anti-inflammatory activity of aqueous and methanolic plant extracts was evaluated in the LPS-enhanced leukocyte migration assay. A concentration-dependent inhibitory trend was observed for *Capraria biflora* (**A**), *Chamaemelum nobile* (**B**), *Harpagophytum procumbens* (**C**), and *Schefflera chimbuensis* (**D**). Values are plotted as relative leukocyte migration, RLM (SEM, n = 3 replicates with 10 larvae each) and a value of 0.5 was established as a cut-off for anti-inflammatory activity. ***p<0.001, **0.001<p<0.01, *0.01<p<0.05.

When tested in the LPS-enhanced leukocyte migration assay, aqueous extracts of *C. nobile*, *H. procumbens*, and *C. biflora*; and methanolic extracts of *S. chimbuensis* showed concentration-dependent anti-inflammatory activity. These effects were reflected by the gradual decrease in the migration of leukocytes to the injured tail and therefore by decline in RLM values, as depicted in Figure 4. In addition to the (expected) anti-inflammatory activity of *C. nobile* (RLM 0.48 at MTC) and *H. procumbens* (RLM 0.46 at MTC), our screening results confirmed the anti-inflammatory activity of *C. biflora*. At their MTC, clustering of leukocytes in the damaged zone in the tails of the larvae was significantly reduced and an RLM value of 0.23 was achieved. Interestingly, *C. biflora* is used in traditional medicine in some countries in Latin America for the treatment of diverse inflammatory conditions [28]–[30]. Furthermore, moderate anti-inflammatory activity (60% inhibition at its MTC) was obtained with the extract of *S. chimbuensis*, for which no previous reports on anti-inflammatory activity have been described.

In general, these results demonstrate a dose-dependent anti-inflammatory effect for all the plant extracts tested in our LPS-enhanced assay (Figure 4). In addition, these results confirm the amenability of the model and the ability of the assay to easily screen and detect even mild anti-inflammatory effects in crude extracts of diverse plant species.

## Discussion

Recently, our laboratory has established zebrafish as a biodiscovery platform to screen for novel active molecules, using zebrafish-based assays in particular for the bioassay-guided identification of drug-like natural products [19], [22], [31]–[33] and synthetic compounds [34]. Within this context, main objectives of this study were first to optimize an anti-inflammatory assay in non-transgenic zebrafish, to validate this improved assay with a panel of steroidal and non-steroidal anti-inflammatory drugs, and to determine the utility of this approach to analyze the anti-inflammatory activity of medicinal plants and their bio-active constituents.

The recent application of several transgenic zebrafish lines to the analysis of inflammation in zebrafish – e.g. *fli-1:eGFP* [6], [20], *BACmpx:GFP* [8], *zMPO:GFP* [7], *lysC:DsRED2* [21], and *fms/mpx* [10], [35] – have enabled the live, *in vivo* imaging of the major cell types involved in the inflammatory response and its resolution. One elegant study involved the non-invasive, chemically-induced damage to lateral line neuromast cells in 56-hpf *BACmpx:GFP* and *lysC:DsRED2* zebrafish larvae, showing that leukocytes localize in clusters along the damaged sensory hair cell population in the trunk and tail of the larvae [21]. Although there are clear advantages of these transgenic zebrafish strains for inflammation research, there are various considerations e.g. intellectual property constraints – that may restrict the widespread use of these tools for drug discovery applications. Additional limitations of this more advanced transgenic approach include the requirement for access to fluorescence imaging equipment and software. For many laboratories, especially those with limited financial resources, applications of zebrafish assays will require the use of cost-efficient assays and protocols, wherein some limitations in screening throughput will be acceptable to achieve this. For this reason, a non-transgenic assay based on the dual activation of the innate immune response combined with a whole-mount *in situ* cytochemical staining method in zebrafish larvae may be an attractive option for the rapid *in vivo* characterization of novel anti-inflammatory compounds of natural origin.

Although it has been shown that a mechanically induced wound triggers the rapid recruitment of leukocytes in zebrafish [8], [12], several improvements to this method were necessary to render it suitable for screening purposes in the context of a natural product discovery effort. For instance, activation of the myeloid linage by transection of the larval tail [8], [12] could be enhanced by exposure to Gram-negative endotoxins. Studies have shown that complete activation of macrophages in 30 hpf embryos is achieved by injection of large doses of either Gram-negative (*Escherichia coli*) or Gram-positive (*Bacillus subtilis*) live bacteria [15]. However, the injection of bacterial suspensions into embryos is time-consuming and requires technical expertise. In contrast, the simple immersion of larvae in solutions of LPS induces an enhanced and consistent inflammatory response (Figure 1E and 1F). This response is possibly mediated by the expression of the pro-inflammatory cytokines tumor necrosis factor alpha (TNF-α) and interleukin (IL)-1β, as reported by others [36]–[38]. Moreover, LPS-mediated release of pro-inflammatory cytokines in zebrafish is induced by activation of Toll-like receptors (TLR) [39], [40]. At least 19 TLR variants – particularly two homologues of the human LPS-sensing TLR (TLR4) – and different intracellular adaptor molecules – MyD88, MAL, TRIF, SARM – are encoded in zebrafish and are already expressed during gastrulation, indicating a strong conservation of this first-line defense mechanism [39]. Interestingly, studies in transgenic zebrafish have revealed that MyD88 is able to trigger TLR-mediated immunity, contributing to essential responses to wounding and bacterial infection [40].

Moreover, we were able to show that migrating leukocytes can be readily visualized with a whole-mount *in situ* staining procedure based on the detection of MPO-positive cells. For this, we used a staining kit (Leucognost® POX, Merck) for MPO activity that uses the substrate 4-chloro-1-naphtol (4-CN) [41], which is less hazardous than the benzidine staining [42] used previously for the *in situ* detection of zebrafish leukocytes [12]. Furthermore, as MPO-positive cells must be scored in the tail tip, we made use of the *nacre* strain to avoid the presence of melanocytes, which at 4 dpf are widely distributed in the larvae and interfere visually in the scoring process. *nacre* (*nac^w2^*) mutants do not express crest-derived melanophores throughout development, however melanin production is normal in the retinal epithelium, suggesting that the mutation is specific to the neural crest and does not affect the synthesis of pigment *per se* [25]. Alternatively, wild-type larvae (in which melanophores first appear at 24 hpf) can be also used in the assay as long as they have been pre-treated with PTU to inhibit melanogenesis [24].

One caveat of this assay is that the simple detection of MPO activity in the tail tip cannot immediately distinguish between the inhibition of leukocyte migration and other mechanisms associated with the resolution of inflammation (i.e. apoptosis or reverse migration of leukocytes). However, by timing the analysis during the peak of the inflammatory response (7 hours post-injury), any reduction in the number of MPO-positive cells in the tail tip is most likely to be due to inhibition of migration.

The validation of the LPS-enhanced leukocyte migration assay in 4 dpf zebrafish larvae was performed with a panel of known anti-inflammatory drugs – both NSAIDs and SAIDs. As expected, the results for each drug showed gradual and significant reduction in the migration of leukocytes towards the site of injury (Figure 3A–E). These data correlate well with other studies showing that anti-inflammatory drugs act as predicted at different points in the inflammatory response in zebrafish [9], [21]. Conversely, when compounds with no known anti-inflammatory activity were tested, no significant influence on leukocyte migration was seen since their RLM values did not approach our established cut-off of 0.5 (Figure 3F–H).

To explore the suitability of the LPS-enhanced leukocyte migration assay, we next used it to test the anti-inflammatory activity of NPs, focusing initially on flavonoids, terpenes, phenolic compounds and iridiods as common, low-molecular weight secondary metabolites responsible for the anti-inflammatory properties of different species in diverse plant genera (e.g. apigenin, luteolin, quercetin, (−)-α-bisabolol in *C. nobile* and harpagoside in *H. procumbens*) [43]–[46]. Consistent with what has been reported in other models, our results showed that all tested NPs displayed a concentration-dependent anti-inflammatory response. (−)-α-bisabolol, apigenin, luteolin, naringenin and quercetin all moderately inhibited leukocyte migration in the LPS-enhanced leukocyte migration assay. Their anti-inflammatory activity might be exerted via various mechanisms of action including inhibition of the production of pro-inflammatory cytokines (IL-1β, IL-6, IL-8 and TNF-α) and cyclooxygenase (COX) and lipoxygenase (LOX) enzymes [47]–[50]. Similarly, caffeic acid and harpagoside exhibited a significant anti-inflammatory response in our assay. Likely, these effects might be also mediated by inhibition of the production of pro-inflammatory cytokines and nitric oxide, as well as for the inhibition of COX enzymes, as demonstrated in other studies [43], [51]–[53]. A comparative vision of the anti-inflammatory effects observed with these NPs in the LPS-enhanced leukocyte migration assay in zebrafish and other *in vivo* and *in vitro* models is given in Table 2.

**Table 2:** Inhibitory effects of some natural products in various inflammatory targets.

Natural product	Zebrafish-based assay		Rodent-based assays		Cell-based assay		References
	MTC	RLM	Target, *model*	Dose	Target, *cell line*	IC_50_	
Apigenin	500 µM	0.35	TNF-α, *C57BL/6J mice* NF-κB, *paw* *edema in mice*	50 mg/kg 25 mg/kg	IL-4, *RBL-2H3* TNF-α, *RBL-2H3* IL-1β, *human monocytes*	3.6 µM 5.3 µM 10 µM	[45], [48], [54]
Luteolin	500 µM	0.41	NF-κB, *paw edema in mice*	50 mg/kg	TNF-α, *RAW 264.7* COX, *rat peritoneal leukocytes*	<1 µM 100 µg/ml	[45], [47], [54]
Naringenin	30 µM	0.37	TNF-α, IL-6, iNOS, COX-2, *rats*	50 mg/kg	TNF-α, *J774.1* PGE_2_, *rat macrophages*	−7.9 µM	[45], [55]
Quercetin	500 µM	0.31	TNF-α, IL-1, *colitic rats*	1 mg/kg	TNF-α, *RAW 264.7* PGE2, *rat macrophages*TNF-α, iNOS, *macrophages*	1 µM 13.9 µM 50 µM	[45], [56]
(−)-α-bisabolol	30 µM	0.48	TNF-α, *paw edema and peritonitis in mice*	200 mg/kg	NO, PGE2, COX-2, *RAW 264.7*	100 µM	[50], [57]
Caffeic acid	500 µM	0.14	IL-1β, *paw edema in mice* IL-1β, IL-6,TNF-α, *mice*	30 mg/kg 2 g/98 gof diet	NO, *RAW 264.7*	4.80 µM	[51], [52], [58]
Harpagoside	300 µM	0.15	COX-1, *paw edema in mice*	50 mg/kg	COX-2, 5-LO, *human whole blood and PMNLs*	–	[53], [59]

In the context of a NP-based drug discovery approach, we also made use of the LPS-enhanced leukocyte migration assay to investigate the anti-inflammatory activity of plants used in traditional medicine. Species with well-documented anti-inflammatory activity such as *C. nobile* [44], [46] and *H. procumbens* [43], [60] were moderately active in our assay at their MTCs (Figure 4). In contrast, crude extracts of *A. montana* and *C. officinalis*, and the essential oil of *M. recutita* were highly toxic for the zebrafish larvae and no anti-inflammatory date could be obtained. These observations led us speculate about the limited indication for oral administration that these samples have, which in the larvae could not be controlled since small molecules are not only taken up by the gills and skin, but also by the gastrointestinal tract.

A significant concentration-dependent anti-inflammatory response was induced by *C. biflora* (Figure 4). These results correlate with other studies in which the aqueous extract of the plant showed a dose-dependent anti-inflammatory effect in *in vivo* models of inflammation (carrageenan-induced paw edema in rats and the peritonitis induced by carrageenan in mouse) [28]. Flavonoids have been suggested as responsible for the anti-inflammatory properties of *C. biflora*. This hypothesis has been, in addition, partially confirmed by our group by the identification of apigenin and luteolin in the aqueous extract of the leaves of the plant (data not shown).

In summary, these data support the further use of this zebrafish-based assay to screen for anti-inflammatory plant extracts, and as shown in a recent published work [22], to contribute to the bioassay-guided fractionation of such extracts for the rapid isolation and quantitative bioactivity analysis of their anti-inflammatory constituents.

## Conclusion

Numerous therapeutic agents are commercially available to treat short and long-term inflammatory diseases. Nevertheless, there is still need for the discovery of novel anti-inflammatory drugs with improved potency and reduced side effects. Natural products will play a key role in this regard as they represent an attractive source of chemical diversity. A significant percentage of the worldwide population still relies on the use of phytotherapies for the treatment of inflammatory conditions, making medicinal plants used for these purposes an excellent source of potentially anti-inflammatory secondary metabolites. Taking these factors into account, in this study we report the optimization and validation of an acute leukocyte migration method in zebrafish larvae to rapidly assess the *in vivo* anti-inflammatory activity of small molecules of natural origin. In addition to the advantages of a zebrafish-based assay over other *in vivo* models, we further show that this LPS-enhanced leukocyte migration assay is suitable for the analysis of the anti-inflammatory properties of plant extracts and for the identification of their bioactive constituents.
